# Bottom Ash Modification via Sintering Process for Its Use as a Potential Heavy Metal Adsorbent: Sorption Kinetics and Mechanism

**DOI:** 10.3390/ma14113060

**Published:** 2021-06-03

**Authors:** Young-Kyu Hong, Jin-Wook Kim, Hyuck-Soo Kim, Sang-Phil Lee, Jae-E. Yang, Sung-Chul Kim

**Affiliations:** 1 Department of Bio-Environmental Chemistry, Chungnam National University, Daejeon 34134, Korea; hyk102030@naver.com (Y.-K.H.); kin1888@naver.com (J.-W.K.); 2 Department of Biological Environment, Kangwon Nation University, Chuncheon 24341, Korea; kimhs25@kangwon.ac.kr (H.-S.K.); tlsehd77@kangwon.ac.kr (S.-P.L.); yangjay@kangwon.ac.kr (J.-E.Y.)

**Keywords:** bottom ash, modification, heavy metal, sorption, kinetics, precipitation

## Abstract

Heavy metal pollution in the environment is a critical issue, engendering ecosystem deterioration and adverse effects on human health. The main objective of this study was to evaluate heavy metal adsorbents by modifying industrial byproducts. The bottom ash was sintered and evaluated for Cd and Pb sorption. Three adsorbents (bottom ash, sintered bottom ash (SBA), and SBA mixed with microorganisms (SBMA)) were tested to evaluate the sorption kinetics and mechanism using a lab-scale batch experiment. The results showed that the highest sorption efficiency was observed for Cd (98.16%) and Pb (98.41%) with 10% SBA. The pseudo-second-order kinetic model (R^2^ > 0.99) represented the sorption kinetics better than the pseudo-first-order kinetic model for the SBA and SBMA, indicating that chemical precipitation could be the dominant sorption mechanism. This result is supported by X-ray photoelectron spectroscopy analysis, demonstrating that -OH, -CO_3_, -O, and -S complexation was formed at the surface of the sintered materials as Cd(OH)_2_ and CdCO_3_ for Cd and PbO, and PbS for Pb. Overall, SBA could be utilized for heavy metal sorption. Further research is necessary to enhance the sorption capacity and longevity of modified industrial byproducts.

## 1. Introduction

Heavy metal pollution in soil is a concern because of its adverse effects on ecosystems and human health [1,2,3]. Among the heavy metals, Cd and Pb are considered major soil pollutants, owing to their severe toxicity and because they result in the deterioration of food crops [4,5,6]. A high concentration of Cd in the human body can cause interference for calcium metabolism, occurring hypercalciuria, and kidney failure [4]. Moreover, bioaccumulated Pb in the human body can deteriorate nerve systems and cardiovascular systems [6]. Agricultural fields that are highly polluted with heavy metals may increase their bioavailable fractions in soil, which is then transferred to food crops [7,8,9]. Generally, heavy metals are released from the waste of abandoned metal mines [10,11] and emissions from industrial complexes [12]. Spoil, tailing, waste rocks, and mine drainage are the main sources of abandoned metal mines and can cause heavy metal pollution in adjacent environments [13,14,15,16].

Recently, researchers have reported various techniques to remediate heavy metals in the soil, including physical, chemical, and biological methods. Landfilling, surface capping, and encapsulation are representative physical remediation [17], while soil washing, immobilization, and chemical stabilization are the most adopted chemical remediation techniques [18,19]. Biological remediation techniques include phytoremediation and bio-augmentation [20,21,22]. Recently, geopolymerization was introduced to remove heavy metals in the aqueous phase due to low energy consumption for production and high removal efficiency of pollutants [23]. Among others, soil washing techniques, combining physical and chemical processes to remove heavy metals with washing solutions, such as strong acids or chelates, are commonly used [24,25,26,27]. However, this technique has drawbacks, such as being labor-intensive, having expensive process costs, and producing secondary pollutants. To overcome these disadvantages, a coupled extraction and cementation technique was applied to detoxify the wastewater containing a high concentration of Zinc [28]. Biological remediation techniques have the advantages of being eco-friendly and having a low cost; however, lower efficiency and time-consuming remediation processes, compared to the physical and chemical processes, are limitations.

Alternatively, chemical immobilization as an in situ technique has been widely used because of its high efficiency in decreasing the bioavailable fraction of heavy metals in the soil at a lower processing cost [17]. To date, numerous soil amendments have been proposed to reduce the bioavailable fraction of heavy metals, including alkaline products to increase soil pH [29,30,31], oxidation/reduction processes [32,33], and porous or organic materials for the sorption of heavy metals [8,34,35,36,37].

Coal plant byproducts, fly ash, and bottom ash (BA) have also been used as amendments to remediate heavy metals in the environment [38]. Fly ash has been used as an adsorbent material for heavy metals because of its high porosity and large specific surface area, resulting in the high sorption efficiency of soluble heavy metals in soil [39]. However, fly ash in its original form has low efficiency for heavy metal sorption; hence, fly ash should be modified to increase the efficiency of heavy metal sorption in soil. For instance, in the hydrothermal method used to modify fly ash with nano-sized kaoline, a high efficiency of Pb sorption was observed [40,41]. BA was also utilized for heavy metal remediation with modifications, such as manufacturing geopolymers or synthesizing zeolite-type adsorbents [42,43]. Moreover, the sintering process was applied to municipal solid waste incinerator bottom ash for making lightweight aggregate and showed the advantage of high porosity, low particle density, and high compressive strength [44]. In addition, the biosorption method of mixing microorganisms with organic materials has been used for removing heavy metals in the environment. Organic materials such as compost can sorb the heavy metals by reacting surface functional groups such as carboxyl and phenolic etc. [45].

The main objective of this study was to evaluate the heavy metal sorption of modified BA produced from a coal power plant. The original BA was sintered or mixed with microorganisms to increase its sorption capacity. The sorption capacity was evaluated by comparing the sorption isotherms, and related X-ray measurements were conducted to verify the sorption mechanism of heavy metals on modified BA.

## 2. Materials and Methods

### 2.1. Raw Materials and Sintering Process

The BA, sintered BA (SBA), and SBA mixed with microorganisms (SMBA) were acquired from a coal power plant in South Korea. The BA was collected after power generation, and the SBA was manufactured by mixing BA, low-quality unburned carbon (UNC), and dredged sand (DS). Notably, BA:UNC:DS was mixed at a 2:5:3 weight ratio and heated at a temperature of 550–600 °C. After cooling to 25 °C, the material was reheated to a temperature of 1000−1200 °C for sintering. After sintering, size fractions were obtained, and a size of less than 2 mm of SBA was used for the experiment. The manufactured SBA was mixed with humic acid (HA) and microorganism (*Bacillus subtilis* sp.) at the ratio of 8:1:1 (SBA:HA:M) by weight basis to produce SMBA.

### 2.2. Sorbent Property Analysis

The Brunauer–Emmett–Teller technique (3Flex, Micrometrics, Norcross, GA, USA) was used to measure the specific surface area of each sorbent with N_2_ adsorption isotherms. X-ray diffraction (XRD; D8 Discover, Bruker, Billerica, MA, USA) measurements were used to identify the crystalline and non-crystalline properties of each sorbent. To analyze the surface structure and microphotography of each sorbent, field emission scanning electron microscopy (Merlin compact, Zeiss, Oberkochen, Germany) measurements were performed. An X-ray photoelectron spectroscopy (XPS) analysis was conducted with a spectrophotometer (K-Alpha+, Thermo Fisher Scientific, Waltham, MA, USA) using an Al Kα source (1486.6 eV of photons).

For the chemical analysis, pH and electric conductivity (EC) were measured using a pH meter (MP220, Mettler Toledo, Worthington, OH, USA) and an EC meter (S230, Mettler Toledo), respectively, after mixing each sorbent with deionized water at a 1:5 (*w*/*v*) ratio for 1 h. The total nitrogen and total carbon were measured using an elemental analyzer (EA1112, Thermo Fisher Scientific). The temperature in the EA reactor was set to 1000 °C, and the flow rate of the carrier gas (He, O_2_, and air) was maintained at 0.12 L/min.

### 2.3. Heavy Metal Sorption Kinetics and Isotherm Experiment

Sorption kinetic experiments for Cd and Pb were conducted with each sorbent, as described in a previous study [46]. First, 20 g of each sorbent was added to a 250 mL flask containing 100 mL of 200 mg/L Cd (CdCl_2_·2.5H_2_O and Pb (Pb(NO_3_)_2_) solution that was prepared individually. The solution pH was maintained at the range of 5.19−5.25 during the experiment. Each Cd and Pb flask was then shaken at 150 rpm for 24 h until sorption attained an equilibrium. Furthermore, the supernatants were collected at a time interval of 0, 1, 2, 5, 10, 20, 30, 60, 360, 720, and 1440 min and filtered through a 0.45 μm membrane filter. The heavy metal concentration of the filtrate was measured using an inductively coupled plasma optical emission spectrometer (8300 DV Perkin Elmer, Waltham, MA, USA).

The sorption isotherm experiment was conducted with various initial concentrations (50−1000 mg/L) of Cd and Pb. Briefly, 10 g of each sorbent was added to a 250 mL flask containing 100 mL of varying initial concentrations of Cd and Pb. The flask was shaken at 150 rpm for 24 h, filtered through a 0.45 μm membrane filter, and the filtrate was used to measure Cd and Pb concentrations.

The sorption isotherms were modeled using the Langmuir and Freundlich isotherm models. Both isotherm models express the relationship between the mass of sorbed Cd and Pb at a constant temperature per unit mass of each sorbent and the heavy metal concentration in the solution [47]. The Langmuir and Freundlich isotherm models can be expressed as follows.

(1) $$\frac{C_{e}}{q_{e}}=\frac{1}{q_{m}}C_{e}+\frac{1}{q_{m}}b$$

where q_e_ is the total amount of Cd or Pb sorbed on the sorbent at equilibrium (mg/g); C_e_ is the concentration of the solution at equilibrium (mg/L); b is the Langmuir constant (L/mg; q_m_ is the total number of binding sites (alternatively, the maximum amount of Cd or Pb per unit mass of sorbent)); and C_0_ is the initial concentration (mg/L).

(2) $$q_{e}=\frac{\left(C_{0}-C_{e}\right)V}{W}$$

where q_e_, C_0_, C_e_, V, and W represent the metal ion sorption amount per unit mass of sorbent (mg/g), initial metal ion concentration (mg/L), equilibrium metal ion concentration (mg/L), solution volume (L), and mass of sorbent (g), respectively.

### 2.4. Statistical Analysis

The experiment was designed completely randomized, and all measurements were performed in triplicate. A statistical analysis was performed using one-way analysis of variance (ANOVA) with *p* < 0.05 by Tukey test comparing multiple means. All statistical analysis was conducted using SPSS software (version 22.0; SPSS Inc., Chicago, IL, USA).

## 3. Results and Discussion

### 3.1. Physicochemical Properties of Each Sorbent

The physicochemical properties of each sorbent are listed in Table 1. The elemental analysis indicated that the carbon and nitrogen contents increased after sintering in SBA and SMBA, owing to the addition of UNC and DS containing 5–25% of carbon. Alkaline properties were observed for the BA (8.02) and SBA (8.68); meanwhile, acidic properties were observed for SMBA (6.79), as humic acid was added during the SMBA production.

**Figure 1:**
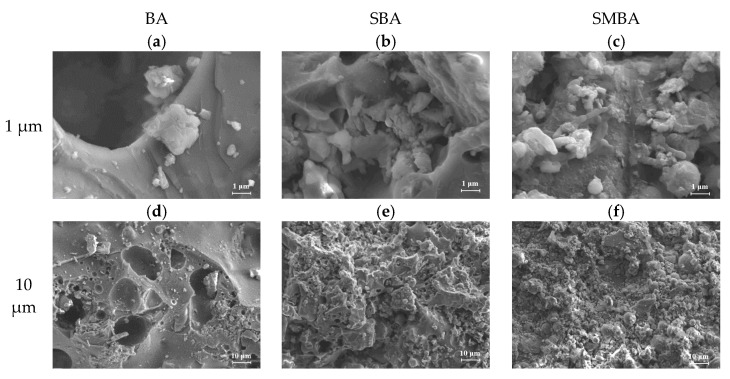
SEM image of absorbents (**a**) BA (1 μm), (**b**) SBA (1 μm), (**c**) SMBAA (1μm), (**d**) BA (10 μm), (**e**) SBA (10 μm), (**f**) SMBA (10 μm).

In terms of the surface area analysis, the surface area of the SBA (7.8477 m^2^/g) was much larger than that of the BA (0.0002 m^2^/g) and SMBA (0.3744 m^2^/g). As shown in Figure 1, the morphology of the BA was sheet-like, whereas the SBA and SMBA exhibited a more porous structure with aggregates of fine crystals and particles on the surface, resulting in a higher surface area. Luo et al. noted that the morphology of BA changed remarkably; a more porous shape was achieved after hydrothermal treatment with the addition of humic acid [48]. The reason for the morphological change in the BA after the hydrothermal or sintering process was the formation of a new crystal phase due to the dissolution of SiO_2_ and Al-containing minerals [49]. Subsequently, Ca-containing minerals can be re-precipitated under alkaline conditions, demonstrating a high sorbent pH [50]. However, the surface area of the SMBA decreased compared to that of the SBA, because the added microorganisms filled the porous area.

**Figure 2:**
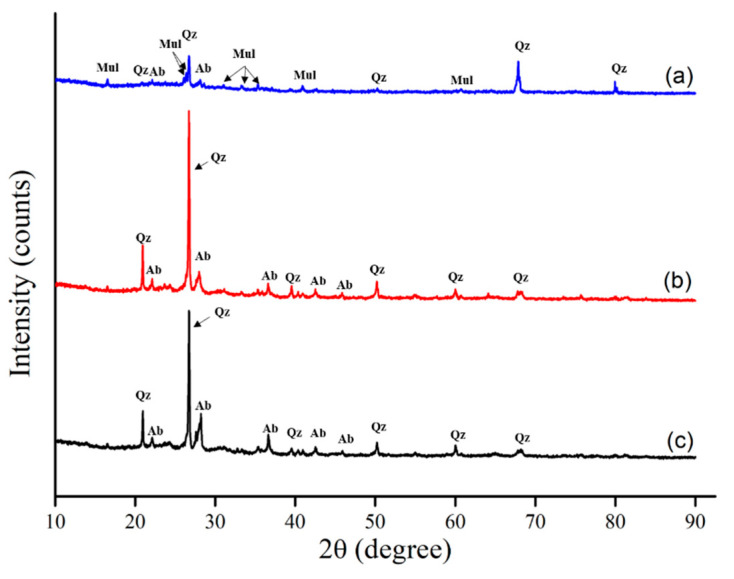
XRD analysis (**a**) BA, (**b**) SBA, (**c**) SMBA (A, M, and Q represents albite, mullite, and quartz).

Figure 2 shows the results of the XRD analysis for the BA, SBA, and SMBA. As shown in the diffractograms, the main crystal phases of the BA are mullite and quartz. After the sintering process, most of the mullite disappeared, and albite was generated. Moreover, an increased intensity of quartz was observed for the SBA and SMBA. This result agreed with a previous study in which the sintering process could alter the formation of crystals on the surface of materials [51]. After the sintering process, carbonate or hydroxide formation was removed, and albite, wollastonite (CaSiO_3_), and quartz (SiO_2_) were the main crystalline phases on the surface of the materials [44]. These pyroxenoid groups, including diopside and wollastonite, are important rock-forming minerals and ultra-basic igneous rocks.

### 3.2. Sorption Efficiency of Absorbent for Cd and Pb Removal

**Figure 3:**
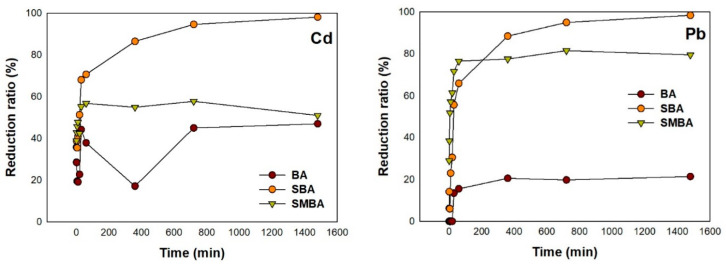
Reduction ratio of Cd and Pb with 3 sorbents at initial concentration of 200 mg/L.

An initial rapid adsorption on all three sorbents was observed for both Cd and Pb, and equilibrium was achieved after approximately 6 h (Figure 3). Among the three sorbents, the sorption efficiency was in the order of SBA > SMBA > BA for both Cd and Pb. The highest adsorption efficiency of Cd and Pb after equilibrium was 98.16% and 98.41%, respectively; for SBA, a reduction rate of 47.03% and 21.38% for Cd and Pb was observed in BA, respectively.

To examine the sorption mechanism, the sorption kinetic data were fitted using a pseudo-first and second-order kinetic model. The fitted kinetic model showed that all three sorbents were better described by the pseudo-second-order kinetic model with an R^2^ value of 0.99, for both Cd and Pb (Table 2). Fan et al. reported that the sorption mechanism could differ based on a fitted sorption kinetic model [46]. When the sorption kinetic model was well fitted to the pseudo-first-order kinetic model, mononuclear sorption could be the main sorption mechanism, whereas chemical sorption could be the main sorption mechanism when pseudo-second-order sorption kinetics data were well fitted. Therefore, in our study, chemical sorption was mainly involved in the adsorption of Cd and Pb on SBA.

The value of K_2_ presented a much higher value for Cd (3.158–6.616) compared to Pb (0.041–0.217) in the pseudo-second-order kinetic model. This result indicated that the affinity of Cd was relatively higher than that of Pb for all three sorbents. Among the three sorbents, the SMBA exhibited the highest k_2_ value for both Cd and Pb, while the maximum adsorption capacity (q_m_^2^) was in the order of SBA > SMBA > BA for both Cd and Pb. This result indicates that, initially, a strong chemical sorption occurred in the SMBA for both Cd and Pb. However, the longevity of the adsorption of Cd and Pb was more efficient with SBA than that with BA and SMBA.

### 3.3. Sorption Isotherms of Cd and Pb

Sorption isotherms generally show the relationship between sorbed compounds (heavy metals in this study) and sorption materials (porous media in this study). Two representative sorption isotherm models (Langmuir and Freundlich) were used to evaluate the sorption capacity of porous media (Table 3 and Figure S1). All the parameters for the BA could not be obtained for both the Langmuir and Freundlich models, indicating a low sorption capacity of BA for heavy metals. For Cd, the maximum sorption capacity using the SBA and SMBA was 0.17 and 0.31 mg/g, respectively; meanwhile, the maximum sorption capacity, using the SBA and SMBA that was calculated for Pb, was 1.93 and 5.30 mg/g, respectively. In the case of the correlation coefficient (R^2^), a much higher correlation coefficient was observed in the Langmuir model than in the Freundlich model, except for the SMBA. This result indicates that the Langmuir isotherm model was more suitable than the Freundlich model for describing the sorption of Cd and Pb using the sorption materials in this study. Fan et al. (2020) noted that the Langmuir isotherm model could determine the sorbed compounds on the surface of sorption materials on the monolayer or equivalent sites, whereas the Freundlich isotherm model describes the adsorption process occurring on a heterogeneous surface [46]. Because a higher correlation coefficient was observed in the Langmuir isotherm model, Cd and Pb were sorbed on the homogeneous surface via monolayer sorption rather than heterogeneous surface sorption with ionic interaction [37].

### 3.4. Sorption Mechanism Evaluation Using XPS Analysis

**Figure 4:**
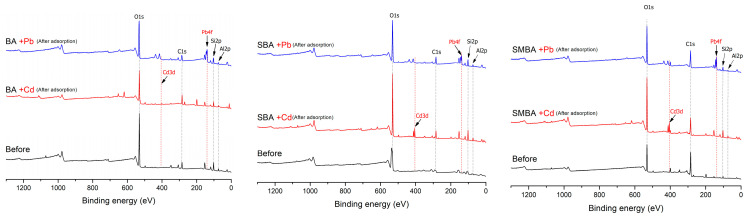
XPS survey spectra for amendments before and after cadmium and lead adsorption.

**Figure 5:**
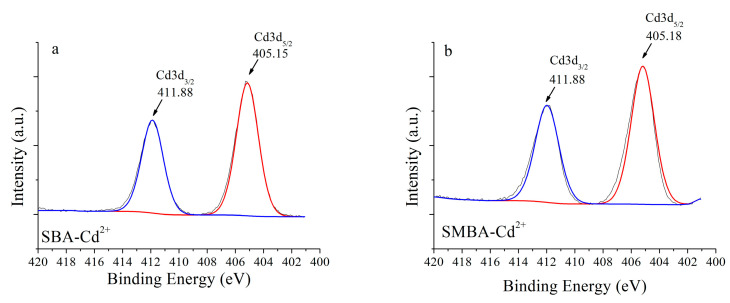
XPS spectra of (**a**) Cd3d for SBA, (**b**) Cd3d for SBMA.

An XPS analysis was conducted to examine the detailed mechanisms of Cd and Pb sorption on porous media. None of the binding spectra of Cd and Pb was observed for the three sorbents before the sorption experiment; however, Cd3d and Pb4f peaks were observed for both the SBA and SMBA sorbents after the sorption experiment (Figure 4). As shown in Figure 5 and Table S2, Cd sorption occurred at Cd3d_3/2_ (411.8 eV) and Cd3d_5/2_ (405.1 eV) for both the SBA and SMBA, indicating that Cd^2+^ ions formed chemical precipitates such as Cd(OH)_2_ and CdCO_3_ [52,53]. The interaction of hydroxide or carbon oxide with Cd^2+^ is straightforward because carboxyl or hydroxyl functional groups can easily react with heavy metal ions on the surface of the sorbent [52].

**Figure 6:**
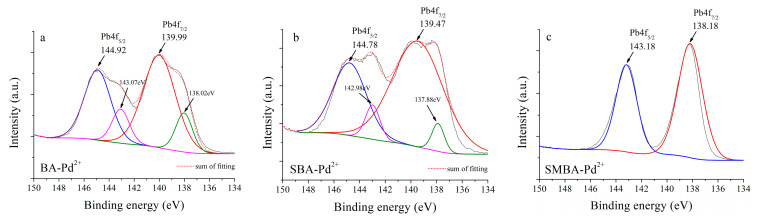
XPS spectra of (**a**) Pb4f for BA, (**b**) Pb4f for SBA, (**c**) Pb4f for SMBA.

As shown in Figure 6, the two peaks at 144.9 and 139.9 eV for BA and at 144.7 and 139.4 eV for SBA were assigned to Pb4f_5/2_ and Pb4f_7/2_, respectively. A previous study reported that the binding energies of 144.7 and 139.4 eV denoted the complexation of PbO, which were in good agreement with our results [46,54]. However, the main binding energy of the SMBA was assigned to 138.18 and 143.18eV, differing by ±1.74−1.29 eV compared to the BA and SBA. These values were reported as PbS complexation for Pb^2+^ ions [55,56]. The difference in binding energy between SMBA and the other two sorbents could be explained by the fact that the SMBA had 2.83% S content on the surface; meanwhile, no S content was observed on the surface of both BA and SBA (Table S1). Sulfur has a high affinity toward heavy metals, and its interaction is generally used to remove ionic forms of heavy metals in solutions or soil [57,58].

## 4. Conclusions

The modification of BA via the sintering process was evaluated for heavy metal sorption. The sintering process altered the morphology of the original BA, increasing the porosity. During the sorption process, initial rapid sorption occurred in the SBA, and equilibrium was attained after 6 h. The highest sorption efficiency with the SBA was achieved at 98.16% for Cd and 98.41% for Pb. The sorption kinetics of Cd and Pb with modified sorbents showed that pseudo-second-order kinetics strongly represented the sorption process, indicating that chemical complexation was the dominant sorption process. This result is also supported by XPS analysis, which revealed that chemical precipitation occurred for Cd, including Cd(OH)_2_ and CdCO_3_. In the case of Pb, the complexation with oxygen (PbO) or sulfur (PbS) occurred on the surface of the SBA. However, no beneficial effect was observed when the microorganisms were mixed with the SBA.

## Figures and Tables

**Table 1:** Physicochemical properties of sorbents.

Heavy Metals	C	N	pH	EC	Surface Area
	%	%		dS/m	m^2^/g
BA	0.30 ± 0.00 ^c^	N.D	8.02 ± 0.61 ^ab^	0.19 ± 0.05 ^c^	0.0002
SBA	4.51 ± 0.04 ^a^	0.05 ± 0.00 ^b^	8.68 ± 0.11 ^a^	1.29 ± 0.03 ^b^	7.8477
SMBA	3.69 ± 0.00 ^b^	0.28 ± 0.00 ^a^	6.79 ± 0.02 ^b^	44.7 ± 1.58 ^a^	0.3744

Different characters (^a, b, c^) are statistically different at *p* < 0.05.

**Table 2:** Sorption kinetic model parameters.

Species	Sorbent	1st Order			2nd Order		
		q_m_^1^ (mg/g)	k_1_ (1/min)	R^2^	q_m_^2^ (mg/g)	k_2_ (1/min)	R^2^
Cd	BA	0.005	0.0009	0.71	0.009	8.549	0.99
	SBA	0.010	0.0025	0.93	0.020	3.158	0.99
	SMBA	0.002	0.0014	0.69	0.012	6.616	0.99
Pb	BA	0.166	0.0025	0.79	0.319	0.116	0.99
	SBA	0.646	0.0035	0.95	1.094	0.041	0.99
	SMBA	0.219	0.0028	0.81	0.914	0.217	0.99

^1^ 1st order kinetics for q_m_^1^; ^2^ 2nd order kinetics for q_m_^2^.

**Table 3:** Sorption isotherm parameters for varied sorption materials.

Heavy Metals		Langmuir			Freundlich		
		Q_max_	b	R^2^	K	1/n	R^2^
		mg/g	L/mg		mg/g		
Cd	BA	-	-	-	-	-	-
	SBA	0.17	0.03	0.91	0.50	0.05	0.60
	SMBA	0.31	0.17	0.93	0.26	0.33	0.64
Pb	BA	0.13	0.14	0.99	0.02	0.57	0.62
	SBA	1.93	0.17	0.99	0.57	0.27	0.72
	SMBA	5.30	0.01	0.64	0.02	0.91	0.93

## Data Availability

The data presented in this study are available on request from the corresponding author.

## Supplementary Materials

The following are available online at https://www.mdpi.com/article/10.3390/ma14113060/s1, Figure S1: Fitting of Langmuir isotherm for Pb sorption with three absorbents, Table S1: Result of EDS for three absorbents, Table S1: Result of XPS with binding energy, FWHM, Chemical state and references.
