# Supplementary material for: Bottom Ash Modification via Sintering Process for Its Use as a Potential Heavy Metal Adsorbent: Sorption Kinetics and Mechanism
Source: Materials (Basel). 2021 Jun 3;14(11):3060. doi: 10.3390/ma14113060 (PMC8200004; doi:10.3390/ma14113060)

Supplementary Data

Table S1. Result of EDS for three absorbents

	BA	SBA	SMBA
		Wt %	
O	52.9	47.1	50.2
Na	0.64	1.18	1.18
Mg	0.89	0.80	0.79
Al	15.2	11.4	12.9
Si	19.8	27.6	21.0
P	0.35	-	0.98
S	-	-	2.83
Cl	-	-	1.90
K	0.80	1.91	1.21
Ca	4.13	2.28	3.30
Ti	1.42	0.77	0.26
Fe	3.89	7.07	3.47
Total	100	100	100

Table S2. Result of XPS with binding energy, FWHM, Chemical state and references

		Binding energy	FWHM (eV)	Chemical	Reference
BA	Cd3d	-	-		
SBA	Cd3d 5/2	405.15	1.84	CdCO3	45
	Cd3d 3/2	411.88	1.88	Cd(OH)2	46
SMBA	Cd3d 5/2	405.18	2.02	CdCO3	45
	Cd3d 3/2	411.88	2.07	Cd(OH)2	46

Figure S1. Fitting of Langmuir isotherm for Pb sorption with three absorbents

		Binding energy	FWHM (eV)	Chemical	Reference
BA	Pb4f 7/2	139.99	3.16	PbO	44, 47
	Pb4f 5/2	144.92	2.57	PbO	44, 47
	Pb4f	138.02	1.64		
	Pb4f	143.07	1.69		
SBA	Pb4f 7/2	139.47	4.88	PbO	44, 47

	Pb4f 5/2	144.78	3.20	PbO	44, 47
	Pb4f	142.98	1.38		

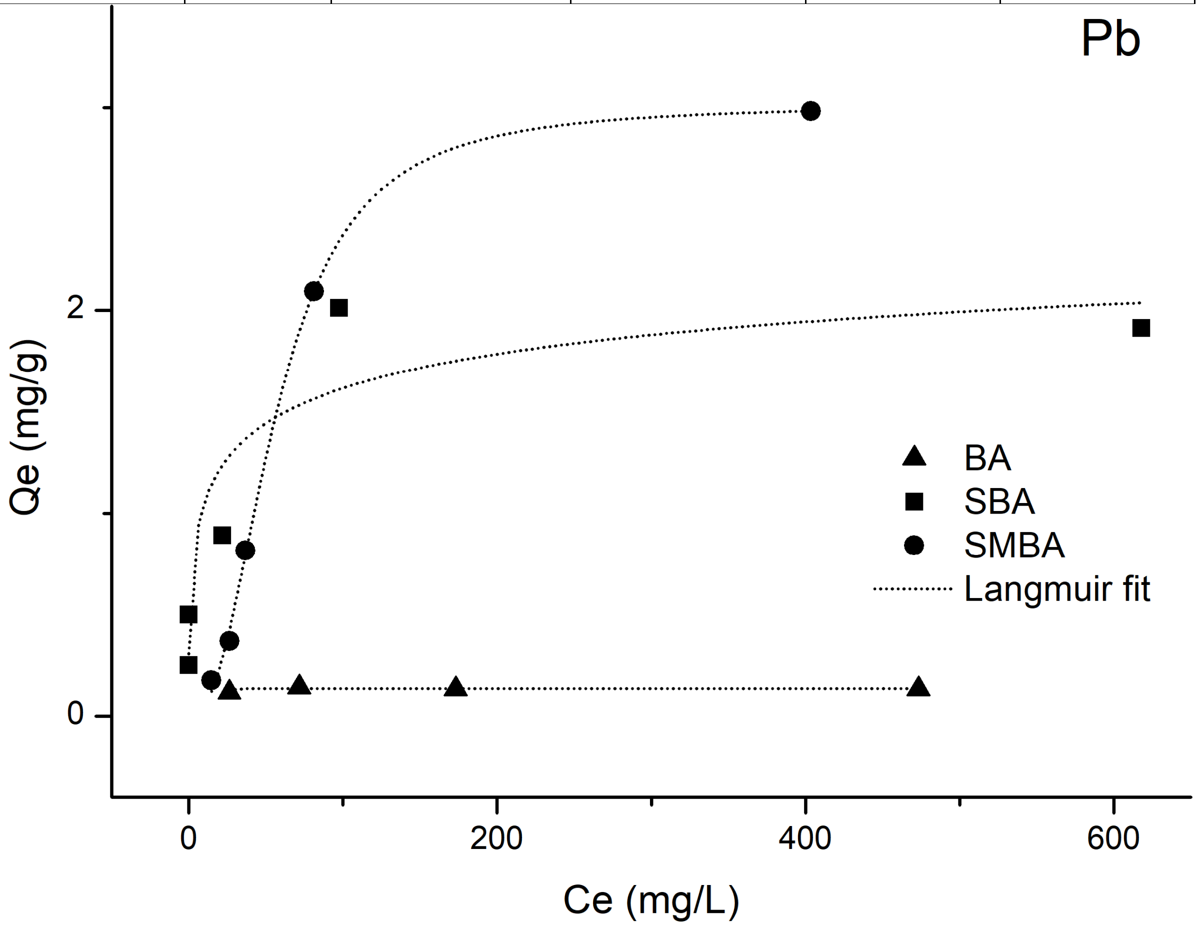

Supplement: Supplementary file 1 [file materials-14-03060-s001.zip › materials-1221763-supplementary.pdf]
